# An Aggressive but Not Malignant Tumor of the Pediatric Hip

**DOI:** 10.5334/jbsr.4149

**Published:** 2026-01-22

**Authors:** Henri Cailliau, Karen Vanslambrouck

**Affiliations:** 1 AZ Sint-Jan, Bruges, Belgium

**Keywords:** pediatric cancer, oncology, desmoid tumor, aggressive fibromatosis

## Abstract

*Teaching point:* Desmoid-type fibromatosis is a rare soft tissue tumor mimicking typical aggressive childhood malignancies, but with inability to metastasize, and with an essential role for imaging for diagnosis and therapeutic strategies.

## Case

**Figure 1:**
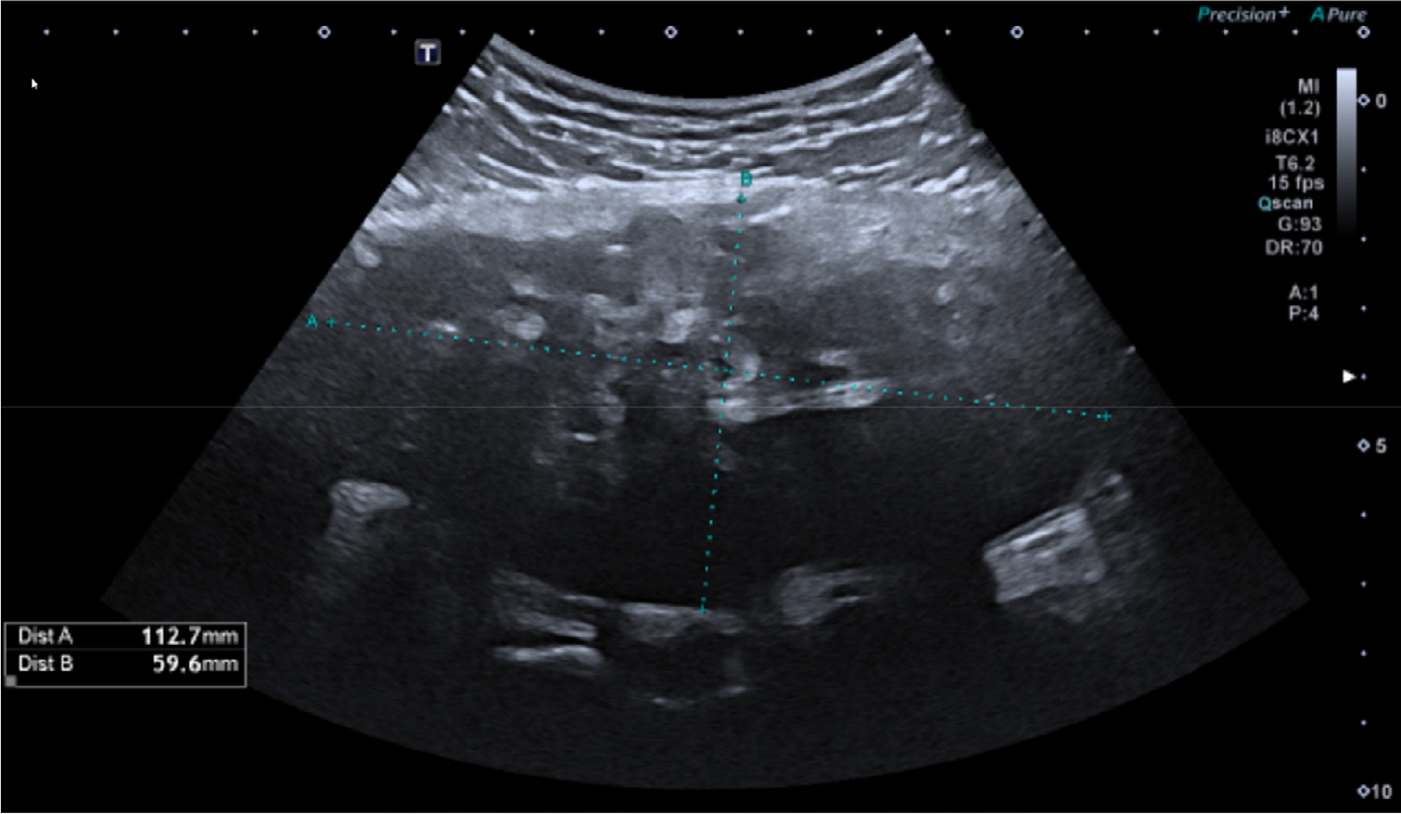
Large heterogeneous intramuscular mass with internal reflections.

A 14-year-old boy of Asian ethnicity presented 3 weeks after a fall with persisting pain and swelling of the right gluteal region. Ultrasound showed a large heterogeneous intramuscular mass with internal reflections and internal vascularity (not shown) (Figure 1).

**Figure 2:**
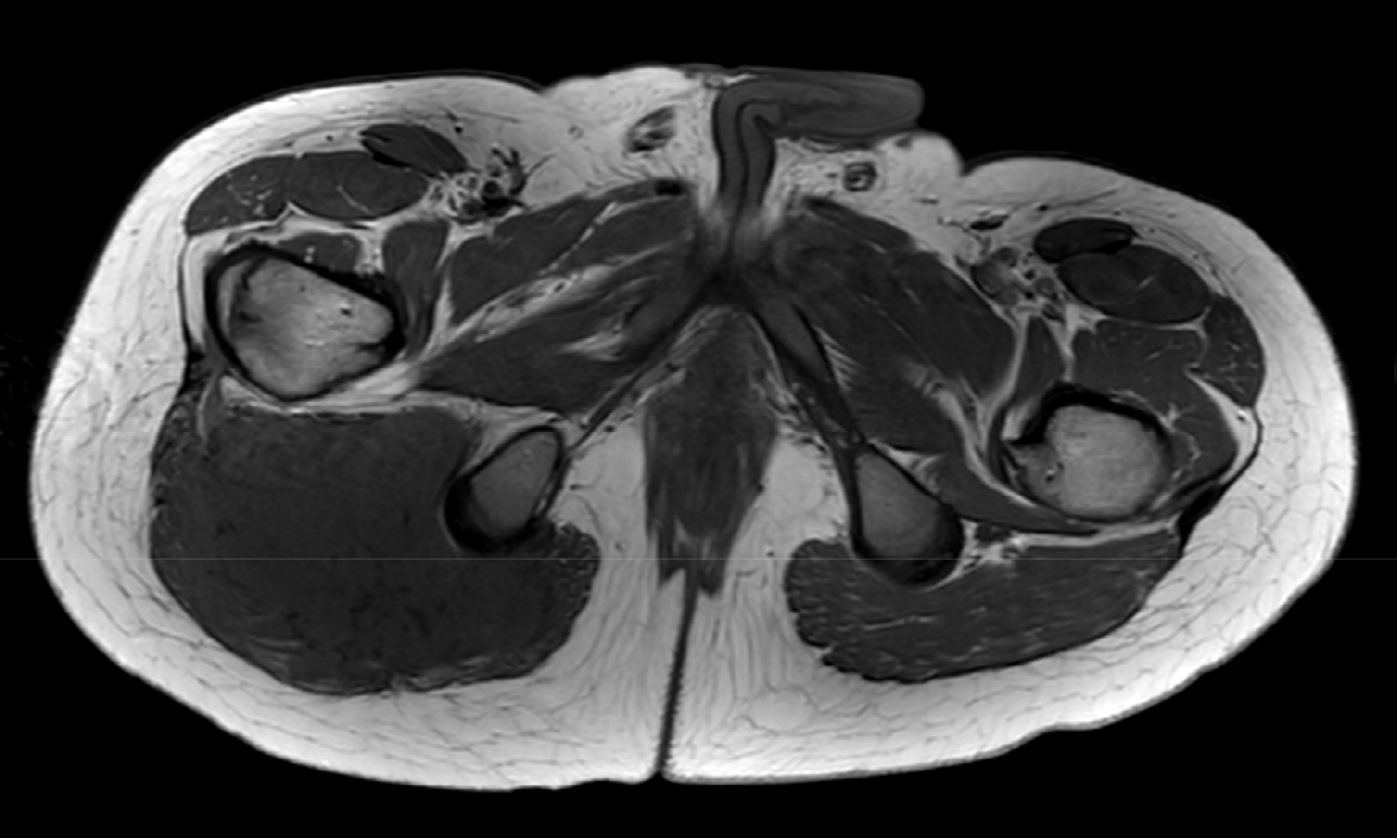
Homogeneous T1-hypointense mass in the right gluteus maximus muscle.

**Figure 3:**
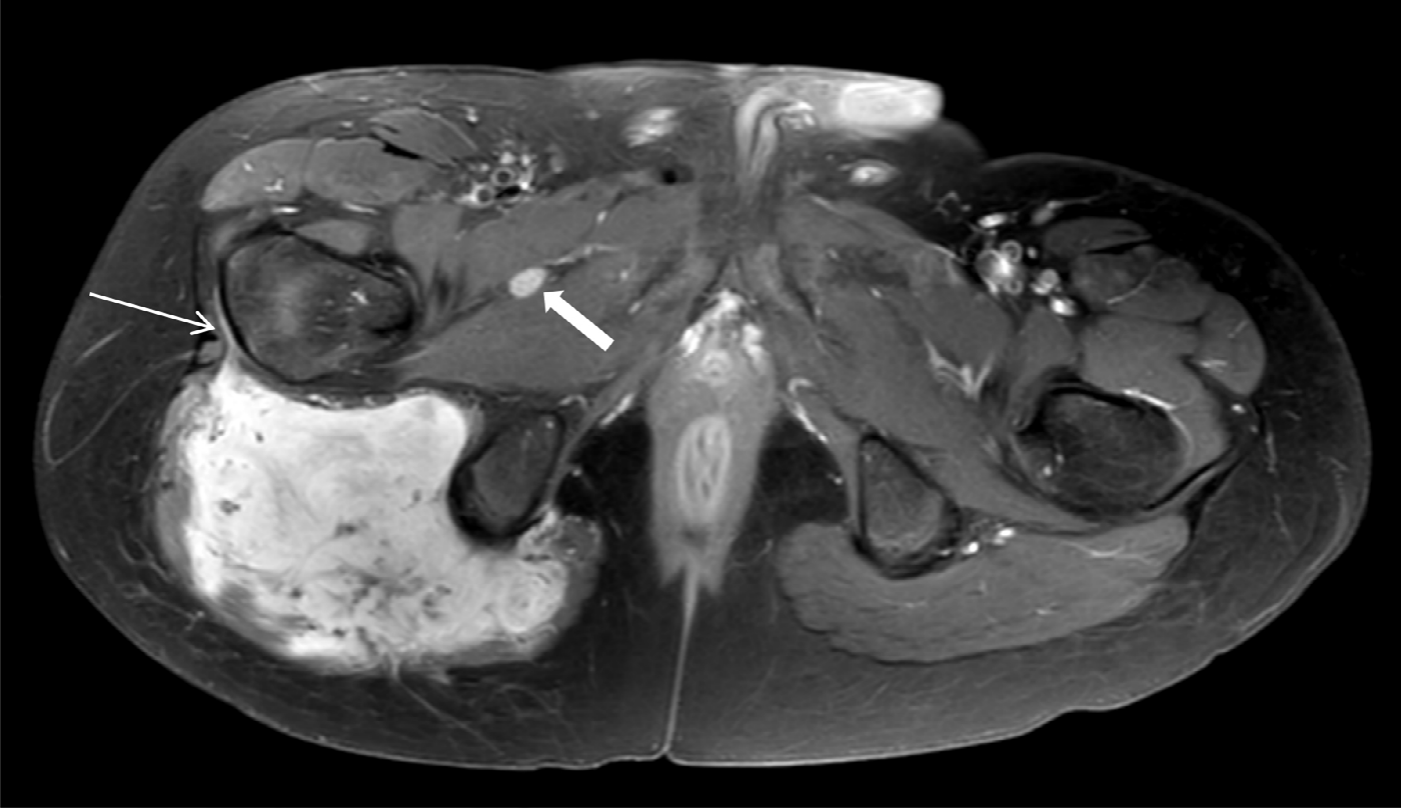
Large vascularised mass in the right gluteus maximus muscle with ‘fascial tail’ at the greater trochanter (thin arrow). Lymfadenopathy between pectineus and adductor muscles (thick arrow).

Magnetic resonance imaging (MRI) confirmed a large intramuscular mass in the right gluteus maximus muscle with displacement of the sciatic nerve, and edema in the surrounding musculature. The tumor showed a homogeneous hypo-intense signal on T1-weighted images (Figure 2). Areas of restricted diffusion (not shown) were noted, as well as vivid contrast enhancement with several internal low-intensity non-enhancing serpiginous structures, and a ‘fascial tail’ at the level of the greater trochanter (Figure 3, thin arrow). Multiple lympadenopathies were observed surrounding the tumor and in the right-sided pelvis (Figure 3, thick arrow).

Computed tomography (CT) of the right hip (not shown) revealed no soft tissue calcifications nor heterotopic ossifications, ruling out pseudo-tumoral alterations seen in myositis ossificans. Tentative diagnosis of an aggressive malignant tumor (most likely rhabdomyosarcoma) was made, and an open biopsy was performed in a tertiary center.

Pathological analysis initially showed findings in keeping with rhabdomyosarcoma; however, molecular analysis and next-generation gene sequencing showed a mutation in the CTNNB1 gene, consistent with the diagnosis of a desmoid-type fibromatosis.

## Comment

Desmoid-type fibromatosis (DF) is a rare soft-tissue tumor with locally aggressive behavior, but inability to metastatic spread. In the pediatric population, the most common location is in the extremities; however, intra-abdominal and abdominal wall tumors also occur. Most cases are sporadic, caused by a pathogenic mutation in the CTNNB1 gene. Aggressive fibromatosis may also be triggered by previous trauma [1]. Both predisposing elements were present in this case.

Preferred imaging modality is MRI, where DF typically shows a T1 hypo-intense signal. There is often vivid contrast enhancement, and a ‘fascial tail’ can sometimes be appreciated, referring to the tapered extension of soft tissue tumor along the fascia. Unenhancing serpiginous bands in the tumor are often seen, corresponding with fibrous bands. Generally, calcifications of hemorrhage are not present.

Differentiation between peripheral nerve sheath tumors or soft tissue sarcomas is crucial for further diagnostic workup and treatment. This can only be made through pathological and molecular analysis.

Treatment options vary depending on symptoms and the tumor’s growth. Given the inability to metastasize, a conservative strategy is often the first approach. Surgery may be recommended for tumors in certain locations that do not respond to observation. In selected cases, radiation of systemic therapy can also be considered in the treatment plan.
